# The short-term effect of bariatric surgery on non-invasive markers of artery function in patients with metabolic syndrome

**DOI:** 10.1186/s13098-015-0076-6

**Published:** 2015-09-16

**Authors:** Justyna Domienik-Karłowicz, Wojciech Lisik, Zuzanna Rymarczyk, Olga Dzikowska-Diduch, Andrzej Chmura, Urszula Demkow, Piotr Pruszczyk

**Affiliations:** Department of General Medicine and Cardiology, Medical University of Warsaw, Lindley’a 5, 02-005 Warsaw, Poland; Department of General Surgery and Transplantology, Medical University of Warsaw, Nowogrodzka 59, 02-005 Warsaw, Poland; Department of Laboratory Medicine and Clinical Immunology of Developmental Age, Medical University of Warsaw, Marszałkowska 24, 00-576 Warsaw, Poland

**Keywords:** Morbid obesity, Bariatric surgery, Body mass index, Flow-mediated dilatation, Pulse wave velocity, Endothelial dysfunction

## Abstract

**Background:**

An improved understanding of the vascular function, measured in non-invasive way, in constantly growing group of patients at increased risk of cardiovascular events is necessary. To evaluate the effects of metabolic syndrome in morbidly obese patients and body mass reduction secondary to gastric bypass surgery on convenient and new non-invasive markers of artery function: pulse wave velocity (PWV), flow- and nitroglycerin-mediated dilatation (FMD, NTG).

**Methods:**

There were 40 patients included into prospective study, who were qualified for bariatric surgery (OB1) and evaluated again 6 m after surgery (OB2). A control group (CG) consisted of 15 healthy women. A second control group (CG2) consisted of 15 women with grade 1 obesity. PWV, FMD, NTG were assessed.

**Results:**

The reduction of BMI (kg/m^2^) from 47.73 ± 6.18 (OB1) to 35.22 ± 5.20 (OB2) was observed. The PWV turned out to be higher before bariatric surgery (OB1 vs. OB2 8.53 ± 1.76 vs. 7.82 ± 1.49 m/s; p < 0.001), however it was no different than PWV in CG. In OB1 group PWV showed correlation with age (r = 0.492, p = 0.001), HR (r = 0.324, p = 0.04), %FM (r = 0.328; p = 0.039), NTG% (r = −0.332, p = 0.036) as well as hsCRP (r = 0.394, p = 0.014). A multivariate analysis showed that the most significant factors influencing PWV were age (p = 0.0005) and hsCRP (p = 0.0014), pseudo R^2^ index 0.44365. The values of FMD differed between OB1 and OB2 groups (12.83 ± 5.15 vs. 17.52 ± 5.50 %; p < 0.0001), however, they were similar to results obtained in CG (14.45 ± 6.14 %; NS). The values of nitroglycerin-mediated dilatation differed between OB1 and OB2 groups (21.47 ± 8.31 vs. 28.54 ± 8.16 %; p < 0.0001) and were lower as compared with CG (31.42 ± 5.95 %; p = 0.0005).

**Conclusion:**

Body mass reduction secondary to bariatric surgery in patients with severe obesity and metabolic syndrome results in improvement of functional markers of artery function and advantageous metabolic changes. The improvement in functional markers of artery function (NTG%) was correlated with change in triglyceride blood concentration.

## Background

According to World Health Organization obesity is an abnormal fat accumulation in the body that may impair health [1, 2]. The most commonly used obesity classification depends on body mass index (BMI), where BMI ≥40 kg/m^2^ indicates severe obesity.

The severe obesity and metabolic syndrome were, until recently, a medical problem concerning only rich countries, but the latest studies have shown that the problem is also present in developing countries in Asia and almost worldwide. An overweight results in a significant increase in the risk of chronic-degenerative diseases and death [3]. One should underline that body mass increase results in elevated risk of one or more diseases from the above mentioned group [1]. A meta-analysis of 15 prospective studies, which included 258 114 patients, showed that an increase in waist circumference of 1 cm leads to the elevation of the risk of cardiovascular disease of 2 %, while an increase of WHR by 0.01, results in cardiovascular disease risk being elevated by 5 % [4].

Bariatric surgery is currently an established treatment method; an part of complex and interdisciplinary therapeutic approach to patients with severe obesity and metabolic syndrome [5]. The indications for bariatric surgery in patients aged 18–65 years include BMI ≥40 kg/m^2^ or BMI 35–40 kg/m^2^ with co-morbidities, the severity of which is expected to decrease with body mass reduction [5].

Vascular remodeling assessment is non-invasive, repeatable, and relatively not expensive [6]. The results of measurements often correlate with the presence of cardiovascular risk factors [7] and arteriosclerosis and allow to predict the risk of fatal and non-fatal cardiovascular events [6, 8]. They are recommended by guidelines to be the early markers of vascular damage, including: pulse wave velocity (PWV), flow-mediated dilatation (FMD), and nitroglycerin-mediated dilatation (NTG) [9–11].

### Objectives

The objective of this study was to determine whether severe obesity in women with metabolic syndrome leads to functional remodeling of arterial wall and change in the corresponding markers. At the same time we assessed the effect of body mass reduction on the above mentioned markers following bariatric surgery.

## Methods

To the prospective study, we recruited 40 premenopausal, morbidly obese women undergoing qualification for bariatric surgery. They were re-assessed 6 months after the bariatric procedure. The inclusion criteria were as follows: severe obesity (BMI >40 kg/m^2^), metabolic syndrome, age >18 and <60 years, female sex. The exclusion criteria were: type 2 diabetes mellitus, history of myocardial infarction, clinically significant valvular heart disease, chronic kidney disease, chronic liver disease, chronic obstructive pulmonary disease, smoking (>20 cigarettes/day), polytherapy, obstructive sleep apnea. The control group consisted of 15 people of middle age, 36 ± 8.34 years, and average body weight of 60.72 ± 5.12 kg. The patients in the control group were matched for sex and age. In all patients included in the study, physical examination and medical interview as well as basic laboratory tests, electrocardiography and PWV, FMD, NTG assessments were performed.

The evaluation of brachial artery vasodilation was performed in accordance with “Guidelines for the ultrasound assessment of endothelial-dependent flow-mediated vasodilation of the brachial artery: a report of the International Brachial Artery Reactivity Task Force” [12]. Thus, in every patient all measurements of FMD and NTG were performed under fasting conditions, after at least 12 h without smoking and withholding treatment with any vasodilators. The examination was performed twice (before and 6 months after surgery), in accordance with a protocol in supine position. The brachial artery was subjected to imaging in the 2D longitudinal plane, using Philips iE 33 system device (Andeouver, Massachusetts, USA) with a 5–7 MHz linear transducer.

Pulse wave velocity was measured in all patients in accordance with the established protocol. The PWV evaluation was performed after night rest according to the European Society of Cardiology guidelines, using Complior SR device (Artech Medical, Paris, France), equipped with two TY-306 tonometric sensors detecting blood pressure variability in the range of 0.01–100 Hz, which includes harmonic frequencies of pressure wave generated by variable heartbeat rate. When the impulses were detected, registered and processed by a computer, the signal was enhanced, in order to obtain pulse wave curve from carotid and femoral artery. Next, a transit time delay between the two pressure waves was automatically calculated, as well as mathematic estimation of the velocity of pulse wave propagation (according to equation: distance between the points of measurements on the body surface/transit time delay) was performed. The study protocol was approved by the Local Bioethics Committee.

### Statistical analysis

The quantitative variables were presented using standard descriptive statistics: means, standard deviation for normally distributed variables, medians and ranges for not-normally distributed variables. For quantitative variables a hypothesis of their normal distribution was tested using a Shapiro–Wilk test and a graphical Q–Q plot. When the analyzed variable was distributed normally, a hypothesis of an equal difference in means in both data sets was tested using a *t* test. While comparing two groups showing not normal distribution of analyzed characteristic, a Wilcoxon test for two independent samples was used. In the analysis of quantitative parameters assessed during the treatment, the respective tests for paired samples were used: the t-test for normally distributed variables and the Wilcoxon test for variables showing distribution other than normal. In correlation analysis of quantitative variables, a Pearson correlation coefficient was used for normally distributed variables, while variables showing other kind of distribution were analyzed using a Spearman correlation coefficient. The association between categorical variables was tested in the set of contingency tables, using a Chi squared test or a Fisher’s exact test, if expected values in table cells were not high enough (i.e. above 5). In the analysis of categorical parameters during the treatment, a McNemar’s test of symmetry for two related samples was used. In the analysis of the influence of selected factors on analyzed parameters (PWV, FMD%, NTG%), the multidimensional, generalized linear models (GLMs) were used. In GLMs the optimal type of link function and error distribution were assigned. The optimal model was selected using stepwise variable selection strategy. As the first model selection criterion, a minimal value of Akaike’s Information Criterion was used. The selected model, being the best fit for the data, was described using the Pearson Chi Squared and pseudo R-Squared statistics. The assumed level of statistical significance was p < 0.05. All calculations were performed using SAS9.2 System.

## Results

### General clinical characteristics of patients with severe obesity and control group

There were 40 premenopausal female patients with severe obesity in the middle age, 36.4 ± 9.0 years; recruited into the study and 15 female patients recruited into the control group (36 ± 8.3 years vs. 36.4 ± 9.0 years; NS). Every bariatric surgery was performed by the same team of surgeons. All patients with severe obesity were reassessed at 6 months after surgery in order to undergo post-operative evaluation.

Anthropometric data of patients were summarized in Table 1.

**Table 1:** Anthropometric data of patients qualified for bariatric surgery and of the control group

	OB1 (n = 40)	Control group (n = 15)	p
Body weight (kg)	132.03 ± 18.42	60.73 ± 5.12	<0.0001
BMI (kg/m^2^)	47.73 ± 6.18	21.61 ± 1.41	<0.0001
Fat (%)	49.15 ± 3.83	25.13 ± 4.88	<0.0001
Fat mass (kg)	65.25 ± 12.81	45.37 ± 3.7	<0.0001
BW (kg)	72.34 ± 16.75		

Values are presented mean ± SD

*BMI* body mass index, *EBW* excess body weight

There were 40 (100 %) patients with hypertension in the study population (n = 40). During the course of the study, 30 (75 %) patients received 2 or more antihypertensive drugs (including angiotensin converting enzyme inhibitors and diuretics); and 10 (25 %) patients used only one antihypertensive agent (angiotensin converting enzyme inhibitor). In 11 (27.5 %) patients, prediabetes was diagnosed, including 6 (15 %) patients with impaired fasting glucose (IFG) and 7 (17.5 %) patients with impaired glucose tolerance (IGT) [13]. In 2 (5 %) patients both of the above mentioned abnormalities were observed simultaneously. The patients with diagnosed type 2 diabetes mellitus were excluded from the study. Disorders of lipid metabolism were present in 22 (55 %) patients and obstructive sleep apnea of at most moderate severity was diagnosed in 8 (20 %) patients.

### Evaluation of basic biochemical parameters in patients with severe obesity and in the control group

Basic biochemical parameters of patients with severe obesity and metabolic syndrome s well as in the control group were summarized in Table 2.

**Table 2:** Evaluation of basic biochemical parameters in patients with severe obesity and metabolic syndrome and in the control group

	OB1 (n = 40)	CG (n = 15)	p
Fasting glucose (mg/dl)^a^	89; 67–124	90; 81–102	NS
Fasting insulin (µIU/ml)^a^	14.07; 5.45–56.33	6.81; 3.57–12.73	<0.0001
HOMA^a^	3.31; 1.14–11.68	1.61; 0.78–3.02	<0.0001
Glucose level 2 h after OGTT (mg/dl)^a^	109.58 ± 31.16	95.27 ± 24.14	NS
Insulin level 2 h after OGTT (µIU/ml)^a^	46.53; 6.23–273.1	19.22; 7.21–52.56	0.001
HbA_1_C (%)^a^	5.65; 5.20–10.90	5.60; 5.30–5.80	0.001
Creatinine (mg/dl)^b^	0.76 ± 0.16	0.77 ± 0.08	NS
Total cholesterol (mg/dl)^b^	199.15 ± 35.13	184.07 ± 28.68	NS
HDL-chol (mg/dl)^b^	52.55 ± 10.97	69.53 ± 10.36	0.001
LDL-chol (mg/dl)^b^	120.30 ± 31.59	102.07 ± 25.32	0.04
Triglycerides (mg/dl)^b^	124.78 ± 47.70	63.80 ± 41.02	0.001
hsCRP (mg/l)^b^	10.19 ± 6.92	0.93 ± 0.76	NS
GFR (ml/min)^b^	96.14 ± 20.76	91.97 ± 16.19	NS

^a^Median; range

^b^Mean ± SD

### Evaluation of functional vascular markers in patients with severe obesity and in the control group

The results of comparative analysis of markers of artery function in patients qualified for bariatric surgery and in the control group were summarized in Table 3.

**Table 3:** Comparison of markers of artery function in patients qualified for bariatric surgery and in the control group

	OB1	CG	p
PWV (m/s)			
Mean ± SD	8.53 ± 1.76^b^	7.98 ± 1.24^b^	NS
Median; range	8.45; 5.30–14.40^a^	7.77; 5.30–10.55^a^	
FMD (%)			
Mean ± SD	12.83 ± 5.15^b^	14.45 ± 6.14^b^	NS
Median; range	12.29; 1.80–21.84^a^	15.77; 3.89–23.80^a^	
NTG (%)			
Mean ± SD	21.47 ± 8.31^b^	31.42 ± 5.95^b^	0.0005
Median; range	21.72; 3.52–40.78^a^	32.48; 20.22–42.69^a^	

*PWV* pulse wave velocity, *FMD* flow-mediated dilatation, *NTG* nitroglycerin-mediated dilatation

^a^Median; range

^b^Mean ± SD

In severely obese patients the tendency to nitroglycerin-mediated vasodilation (21.47 ± 8.31 % vs. 31.42 ± 5.95; p < 0.05) was lower. There were no differences between study populations in mean values of pulse wave velocity and flow-mediated dilatation.

### Changes in values of anthropometric parameters in patients with severe obesity

A comparison of basic anthropometric and biochemical parameters in patients before and 6 months after bariatric surgery was presented in Table 4.

**Table 4:** Comparison of basic anthropometric and biochemical parameters in patients before (OB1) and 6 months after (OB2) bariatric surgery

	OB1 (n = 40)	OB2 (n = 40)	p
Body weight (kg)	132.03 ± 18.42	97.46 ± 15.35	<0.0001
BMI (kg/m^2^)	47.73 ± 6.18	35.22 ± 5.20	<0.0001
FAT (%)	49.15 ± 3.83	38.20 ± 5.66	<0.0001
FFM (kg)	66.78 ± 7.84	59.90 ± 8.92	<0.0001
EBW (kg)	72.34 ± 16.75	37.77 ± 13.97	<0.0001
Fasting glucose (mg/dl)	90.40 ± 10.19	85.35 ± 9.50	0.0139
Fasting insulin (µIU/ml)	17.68 ± 11.29	13.26 ± 20.99	<0.0001
HOMA	3.89 ± 2.33	2.79 ± 4.53	<0.0001
Glucose level 2 h after OGTT (mg/dl)	109.58 ± 31.16	80.20 ± 20.18	<0.0001
Insulin level 2 h after OGTT (µIU/ml)	60.72 ± 53.92	13.77 ± 10.64	<0.0001
HbA_1_C (%)	5.80 ± 0.89	5.37 ± 0.30	<0.0001
Creatinine (mg/dl)	0.76 ± 0.16	0.77 ± 0.12	NS
Total cholesterol (mg/dl)	199.15 ± 35.13	189.75 ± 29.64	<0.0001
HDL-chol (mg/dl)	52.55 ± 10.97	54.50 ± 9.68	NS
LDL-chol (mg/dl)	120.30 ± 31.59	113.25 ± 28.42	NS
Triglycerides (mg/dl)	124.78 ± 47.70	94.58 ± 40.82	<0.0001
hsCRP (mg/l)	10.19 ± 6.92	4.76 ± 5.62	<0.0001
GFR (ml/min)	96.14 ± 20.76	93.69 ± 19.30	NS

It is worth noticing that in the above comparison hyperinsulinemia was observed in 19 (47.5 %) patients in OB1 group and in 6 (15 %) patients in OB2 group. The index HOMA values of insulin resistance showed statistically significant differences between the groups (1.76; 0.35–24.80 vs. 3.31; 1.16–11.68; p < 0.0001). The insulin resistance was diagnosed in 26 (67 %) patients in OB1 group and in 9 (22.5 %) patients in OB2 group. In 18 patients 6 months after the surgery, the parameters diagnostic for insulin resistance got normal. HbA_1_C results showed statistically significant differences between the groups (5.35; 4.70–6.10 vs. 5.65; 5.20–6.60; p < 0.0001). There were also statistically significant differences found in the total cholesterol, triglycerides and hsCRP concentrations. There were no differences in creatinine, HDL cholesterol and LDL cholesterol concentrations, as well as in GFR results.

### Evaluation of markers of artery function in patients with severe obesity before and after surgery (OB1 vs. OB2)

A comparison of markers of artery function in patients in OB1 and OB2 groups was presented in Table 5.

**Table 5:** A comparison of markers of artery function in patients before and 6 months after bariatric surgery

	OB1	OB2	p
PWV (m/s)	8.53 ± 1.76^b^	7.82 ± 1.49^b^	0.0226
	8.45; 5.30–14.40^a^	7.63; 4.58–10.99^a^	
FMD (%)	12.83 ± 5.15^b^	17.52 ± 5.50^b^	<0.0001
	12.29; 1.80–21.84^a^	17.22; 6.30–27.41^a^	
NTG (%)	21.47 ± 8.31^b^	28.54 ± 8.16^b^	<0.0001
	21.72; 3.52–40.78^a^	26.92; 13.86–48.63^a^	

*PWV* pulse wave velocity, *FMD* flow-mediated dilatation, *NTG* nitroglycerin-mediated dilatation

^a^Median; range

^b^Mean ± SD

There were statistically significant differences observed in the values of all investigated parameters (PWV, FMD, NTG).

### Correlations

In the group of patients with normal body weight, a negative correlation between body fat mass and pulse wave velocity (r = −0.49, p = 0.063) of borderline statistical significance was observed. Additionally there were the following correlations observed: between PWV and: age (r = 0.49; p = 0.06), FM (kg) (r = −0.49; p = 0.06), heart rate (r = −0.59, p = 0.02); GFR (r = −0.6; p = 0.0171), creatinine (r = 0.54; p = 0.0372) and HDL cholesterol (r = 0.55; p = 0.0331). In OB1 group a correlation between pulse wave velocity and age (r = 0.49; p = 0.0012) or pulse rate (r = 0.324; p = 0.041) was also observed, likewise in control group. A positive correlation with % of fatty tissue %FM (r = 0.328; p = 0.039) and marker of inflammation hsCRP (r = 0.393, p = 0.014), as well as a negative correlation with nitroglycerin-mediated vasodilation results (r = −0.332; p = 0.036) are worth noticing.

### Changes in investigated parameters 6 months after bariatric surgery

An examination performed 6 months after bariatric surgery revealed decrease in EBW of 34.57 ± 9.71 kg. A change in NTG values (ΔNTG%) was correlated with change in triglycerides concentration (r = 0.343, p = 0.04).

Following analysis of the influence of particular variables on PWV value, the following variables were considered as the most significant among the investigated parameters: age (p = 0.0005) and hsCRP (p = 0.0014). The value of a deviance (Pearson Chi Squared) was 0.10 and the value of pseudo-R^2^ was 0.44365.

Following analysis of the influence of particular variables on FMD% it turned out, that the best model fit involved substantial random component (bad fit), which meant that among investigated variables, there were no statistically significant factors influencing the value of analyzed parameter. The best model fit involved the following variables: age (NS) and hsCRP (NS). The value of a deviance (Pearson Chi Squared) was 889.26 and the value of pseudo-R^2^ was 0.02352.

## Discussion

In this paper a non-invasive evaluation of the artery function extend in patients with severe obesity, compared to control group with normal body weight, was performed, as well as assessment of efficiency of surgical treatment for obesity in normalization of the above mentioned parameters. The study population involved 40 female patients in middle age 36.4 ± 9.0 with severe obesity, meeting qualification criteria for bariatric surgery [5]. Patients qualified for bariatric surgery showed no significant age differences in comparison with controls. Comparing the two groups, higher fasting insulin levels and index HOMA values of insulin resistance were found in patients qualified for bariatric surgery. The fasting insulin level exceeding 15 µIU/ml and indicating hyperinsulinemia was observed in 19 (48.71 %) patients with severe obesity and additionally 67 % of patients with severe obesity were diagnosed with insulin resistance. Neither hyperinsulinemia, nor insulin resistance was observed in patients in control group.

In both groups there were no differences in mean values of PWV and FMD%, however, in the group of severely obese patients lower values of NTG% were observed.

Following 6 months of follow-up period after surgery a significant decrease in BMI from 47.73 ± 6.18 kg/m^2^ to 35.22 ± 5.20 kg/m^2^ (p < 0.0001) and in excess body weight (EBW) from 72.34 ± 16.75 kg to 37.77 ± 13.97 kg (p < 0.0001) was observed. A detailed analysis revealed that after 6 months of follow-up the problem of severe obesity was applicable to only 7 out of 40 patients (18 %), while 6 (15 %) patients were classified to category of BMI <30 kg/m^2^ indicating only overweight.

An increased pulse wale velocity is and established risk factor of cardiovascular complications and it is believed to be one of the signs of adverse artery function in patients with hypertension. In the obese patients, both before and after surgery, the PWV values were higher than 12 m/s, which is the upper limit of normal. However, it should be noticed that PWV values in the group of obese patients before bariatric surgery were significantly higher than 6 months after procedure in OB2 group (8.53 ± 1.76 m/s vs. 7.82 ± 1.49 m/s; p = 0.0226). In multidimensional analysis as the most significant variables for predicting PWV values, the following parameters were chosen: age (p = 0.0005) and hsCRP (p = 0.0014), the value of pseudo-R^2^ was 0.44365. The above result is in accordance with the findings of Pirro et al., who showed that CRP concentration is, apart from standard risk factors of cardiovascular diseases, independent predictor of increased vessel stiffness and with the results of the study by Flegall et al., where decrease in vessels elasticity progressing with age and independent from BMI was observed.

To the best of our knowledge in the available literature there are no studies including study populations, which could serve as a reference for our investigated population. Nordstrand et al. in their study in women with severe obesity found on the basis of measurements performed by Sphygmocor device that the mean values of PWV accounted for 8.1 m/s [14].

Flow- and nitroglycerin-mediated vasodilation values in OB1 and OB2 group showed statistically significant differences (12.83 ± 5.15 vs. 17.52 ± 5.50 %; p < 0.0001; and 21.47 ± 8.31 vs. 28.54 ± 8.16 %; p < 0.0001) and were lower than in CG. Kearney et al. in their study in 8 patients, who underwent bariatric surgery, found that mean body mass reduction of 23.4 kg resulted in improvement of FMD from 5.3 to 10.2 %. In the available literature there are no studies, which could be a reference for our study population. Arkan et al. in their study in 203 obese patients found that body weight is a significant predictor of vessels dysfunction. The FMD values correlated with body weight (β = −0.23, p = 0.005) but not with BMI [15]. Benjamin et al. investigated group of patients taking part in Framingham Heart Study and found negative correlation between BMI and FMD [16].

Biasucci et al. described paradoxically good vessels function in patients with severe obesity. Comparing small groups of patients with severe obesity or obesity with people having normal weight, they found higher values of FMD in severely obese patients group than in groups of obese or normal weight patients (13.02 ± 6.50 % vs. 7.53 ± 5.47 %, p = 0.019 and 13.02 ± 6.50 % vs. 7.33 ± 3.68 %, p = 0.011, respectively). The authors did not reveal any difference in FMD between group of normal weight participants and group of severely obese patients (7.33 ± 3.68 % vs. 7.53 ± 5.47 %, NS) [17].

Arkan et al. in their study in 203 obese patients found that body weight is an important predictor of vessels dysfunction. The FMD values correlated with body weight (β = −0.23, p = 0.005) but not with BMI [15]. Benjamin et al. investigated group of patients taking part in Framingham Heart Study and found negative correlation between BMI and FMD [16].

### Study limitations

While discussing study results, its limitations should be considered. During observation in the study, patients continued their medications. In the investigated population antihypertensive drugs were used: enalapril, loop diuretics, calcium channel blockers; the control group denied any drug use. Considering potentially high cardiovascular risk in the investigated population, authors did not decide to stop medications before performing study measurements. It is worth noticing that the above mentioned dugs were used also before the first measurements were taken.

## Conclusions

In summary one should notice that non-invasive evaluation of functional markers of artery function and metabolic indicators implies their negative changes in the group of women with severe obesity as compared with healthy controls. In obese patients following only short (6 months) postoperative period, the improvement of functional markers of artery function and advantageous metabolic changes were observed. The improvement in functional markers of artery function (NTG%) was correlated with change in triglycerides blood concentration. It is worth noticing that in the group of patients with severe obesity and metabolic syndrome, the variables influencing pulse wave velocity were age and hsCRP concentration. Further observation of investigated population is recommended.

## Abbreviations

PWV: pulse wave velocity
FMD: flow-mediated dilatation
NTG: nitroglycerin-mediated dilatation
OB1: the group of patients qualified for bariatric surgery
OB2: the group of patients evaluated again 6 m after surgery
CG: the control group
BMI: body mass index
EBW: excess body weight
GLMs: generalized linear models
